# Antibiotic prescribing for acute gastroenteritis during ambulatory care visits—United States, 2006–2015

**DOI:** 10.1017/ice.2021.522

**Published:** 2022-12

**Authors:** Jennifer P. Collins, Laura M. King, Sarah A. Collier, John Person, Megan E. Gerdes, Stacy M. Crim, Monina Bartoces, Katherine E. Fleming-Dutra, Cindy R. Friedman, Louise K. Francois Watkins

**Affiliations:** 1 Enteric Diseases Epidemiology Branch, Division of Foodborne, Waterborne, and Environmental Diseases, Centers for Disease Control and Prevention, Atlanta, Georgia; 2 Epidemic Intelligence Service, Center for Surveillance, Epidemiology and Laboratory Services, Centers for Disease Control and Prevention, Atlanta, Georgia; 3 Office of Antibiotic Stewardship, Prevention and Response Branch, Division of Healthcare Quality and Promotion, Centers for Disease Control and Prevention, Atlanta, Georgia; 4 Waterborne Disease Prevention Branch, Division of Foodborne, Waterborne, and Environmental Diseases, Centers for Disease Control and Prevention, Atlanta, Georgia; 5 Oak Ridge Institute for Science and Education, Oak Ridge, Tennessee

## Abstract

**Objective:**

To describe national antibiotic prescribing for acute gastroenteritis (AGE).

**Setting:**

Ambulatory care.

**Methods:**

We included visits with diagnoses for bacterial and viral gastrointestinal infections from the National Ambulatory Medical Care Survey and National Hospital Ambulatory Medical Care Survey (NAMCS/NHAMCS; 2006–2015) and the IBM Watson 2014 MarketScan Commercial Claims and Encounters Database. For NAMCS/NHAMCS, we calculated annual percentage estimates and 99% confidence intervals (CIs) of visits with antibiotics prescribed; sample sizes were too small to calculate estimates by pathogen. For MarketScan, we used Poisson regression to calculate the percentage of visits with antibiotics prescribed and 95% CIs, including by pathogen.

**Results:**

We included 10,210 NAMCS/NHAMCS AGE visits; an estimated 13.3% (99% CI, 11.2%–15.4%) resulted in antibiotic prescriptions, most frequently fluoroquinolones (28.7%; 99% CI, 21.1%–36.3%), nitroimidazoles (20.2%; 99% CI, 14.0%–26.4%), and penicillins (18.9%; 99% CI, 11.6%–26.2%). In NAMCS/NHAMCS, antibiotic prescribing was least frequent in emergency departments (10.8%; 99% CI, 9.5%–12.1%). Among 1,868,465 MarketScan AGE visits, antibiotics were prescribed for 13.8% (95% CI, 13.7%−13.8%), most commonly for *Yersinia* (46.7%; 95% CI, 21.4%–71.9%), *Campylobacter* (44.8%; 95% CI, 41.5%–48.1%), *Shigella* (39.7%; 95% CI, 35.9%–43.6%), typhoid or paratyphoid fever (32.7%; (95% CI, 27.2%–38.3%), and nontyphoidal *Salmonella* (31.7%; 95% CI, 29.5%–33.9%). Antibiotics were prescribed for 12.3% (95% CI, 11.7%–13.0%) of visits for viral gastroenteritis.

**Conclusions:**

Overall, ∼13% of AGE visits resulted in antibiotic prescriptions. Antibiotics were unnecessarily prescribed for viral gastroenteritis and some bacterial infections for which antibiotics are not recommended. Antibiotic stewardship assessments and interventions for AGE are needed in ambulatory settings.

Acute gastroenteritis (AGE) causes ∼179 million episodes of acute diarrhea each year in the United States.^1^ Annually, ∼75 million diarrheal illnesses result in visits to US healthcare providers, primarily in outpatient settings.^2,3^ Given that self-limited viral and bacterial illnesses are the most common causes of acute gastroenteritis,^4^ antibiotics are not recommended for most patients.^5^ Unnecessary antibiotic use contributes to antibiotic resistance, which has been increasing among enteric and other pathogens.^6^

Better understanding antibiotic prescribing practices for enteric infections could inform antibiotic stewardship efforts. National antibiotic prescribing for AGE has not been well described. Studies in select populations have reported antibiotic prescribing rates of 10%–25% for acute diarrhea.^2,7^ We describe rates of antibiotic prescribing for AGE in the United States.

## Methods

### Data sources

We used 2006–2015 National Ambulatory Medical Care Survey (NAMCS) and National Hospital Ambulatory Medical Care Survey (NHAMCS) data and a comparable year (2014) of data from the IBM Watson MarketScan Commercial Claims and Encounters Database (henceforth “MarketScan”). Study periods were chosen to predate adoption of *International Classification of Diseases, Tenth revision, Clinical Modification* (ICD-10-CM) codes. We chose NAMCS and NHAMCS data for their national representativeness. We used MarketScan data to describe antibiotic prescribing according to variables for which NAMCS/NHAMCS sample sizes were too small to generate reliable estimates and those not available in NAMCS/NHAMCS.

NAMCS and NHAMCS are national surveys designed and implemented by the National Center for Health Statistics (NCHS). The publicly available data are nationally representative, based on systematic random samples of ambulatory medical care visits, using a multistage probability sampling design.^8^ NAMCS collects data on visits to office-based practices; all specialties except radiology, pathology, and anesthesia are included. NHAMCS collects data on visits to emergency and outpatient departments in nongovernment general and short-stay hospitals. As recommended by NCHS, we excluded NAMCS data from community health centers and NHAMCS data from outpatient departments because they were not available during the entire study period (personal communication).^9^ Both NAMCS and NHAMCS capture patient demographics, physician diagnoses, services, medications, and other treatments.

MarketScan is a convenience sample of deidentified medical claims for persons aged <65 years with private medical insurance.^10^ We used the “outpatient” and “outpatient prescription” tables and defined a visit as the unique combination of enrollee identification number, service date, and service location. During 2014, the sample size was 47 million covered lives.

The National Center for Emerging and Zoonotic Infectious Diseases’ human subjects’ advisor deemed these deidentified NAMCS/NHAMCS and MarketScan data nonhuman subjects; institutional review board review was not required.

### Variables

We defined an AGE visit as one with an *International Classification of Diseases, 9* ^*th*^ *revision, Clinical Modification* (ICD-9-CM) code for ≥1 of the following: bacterial gastrointestinal infection (excluding cholera and *Clostridioides difficile*), viral gastroenteritis, vomiting, or diarrhea (Supplementary Table 1 online). We restricted the NAMCS/NHAMCS analysis to new problems (duration <3 months), captured by the “major reason for the visit” variable. MarketScan does not capture problem duration.

For NAMCS and NHAMCS, we used all available ICD-9-CM codes (up to 3 per visit during 2006–2013; up to 5 per visit during 2014–2015). For MarketScan, we included all diagnosis codes for each claim associated with a single visit. We adapted a previously described tiered algorithm to assign ICD-9-CM codes to tiers based on the likelihood of requiring antibiotics (Supplementary Table 2 online).^11^ Antibiotics are almost always indicated for tier 1 diagnoses. Antibiotics may be indicated for tier 2 conditions, including AGE. Antibiotics are generally not indicated for tier 3 conditions. We excluded AGE visits that included a tier 1 ICD-9-CM code. No NAMCS/NHAMCS AGE visits had another tier 2 code. In MarketScan, 1.9% (n = 36,018) of AGE visits also had an ICD-9-CM code for “other and unspecified noninfectious gastroenteritis and colitis,” which we did not include in our AGE definition. Moreover, <0.25% of MarketScan AGE visits had tier 2 codes other than “other and unspecified noninfectious gastroenteritis and colitis.”

We coded antibiotic prescriptions based on their generic components and therapeutic classes using Lexicon Plus (Cerner Multum, Kansas City, MO, for NAMCS/NHAMCS)^11^ and Micromedex Red Book (IBM Watson Health, Armonk, NY, for MarketScan). For NAMCS/NHAMCS, we extracted antibiotics from data on medications ordered, provided, or prescribed at each visit. Route of antibiotic administration was not available in NAMCS/NHAMCS, and we excluded antibiotics only available in topical formulations. For MarketScan, all available nonparenteral prescription drug claims were linked to the most recent outpatient visit within the preceding 3 days.

We analyzed antibiotic prescribing according to age group (0–4, 5–17, 18–49, and 50–64 years; ≥65 years only available in NAMCS/NHAMCS), sex, visit setting (emergency department and outpatient office settings for both; urgent care and retail clinic settings only available in MarketScan), and ICD-9 codes (NAMCS/NHAMCS sample sizes for viral gastroenteritis and bacterial pathogens were too small to generate reliable estimates). For NAMCS/NHAMCS, we also analyzed antibiotic prescribing according to variables not available in MarketScan: race/ethnicity, insurance type, fever (temperature ≥38.3°C or 100.9°F) at the visit, and whether a complete blood count (CBC) was ordered. For MarketScan, we also analyzed antibiotic prescribing according to variables not available in NAMCS/NHAMCS (stool culture orders) or for which NAMCS/NHAMCS sample sizes were too small to generate reliable estimates (ie, provider specialty). We used current procedural terminology (CPT) codes 87045, 87046, and 87427 to identify MarketScan visits where stool cultures were ordered.

We assessed concordance of antibiotic prescriptions with *2017 Infectious Diseases Society of America Clinical Practice Guidelines for the Diagnosis and Management of Infectious Diarrhea* (“IDSA guidelines”) for conditions with recommended antibiotics.^5^ Antibiotics were considered “concordant” with 2017 IDSA guidelines if the class included a first choice or alternative recommendation. For shigellosis, IDSA guidelines stipulate “if susceptible” for trimethoprim-sulfamethoxazole and ampicillin. We calculated concordance assuming all visits with these antibiotics were either concordant or discordant to determine upper and lower estimates, respectively.

### Statistical analyses

We performed all statistical analyses using SAS version 9.4 software (SAS Institute, Cary, NC). We used 10 years of NAMCS/NHAMCS data to have a sufficient sample for reliable weighted estimates. We calculated national annual percentage estimates and confidence intervals using complex sample methods and sampled visit weights.^11^ We report estimates that met established standards of reliability and precision (ie, those based on ≥30 sampled visits and with a relative standard error <30%).^12^ We compared the weighted mean percentage of visits with antibiotics prescribed between strata using the χ^2^ and an α level of .01, as recommended by the NCHS. For the MarketScan analysis, we used Poisson regression to calculate 95% confidence intervals.

## Results

### Nationally representative estimates (NAMCS/NHAMCS, 2006–2015)

#### Prescribing overall and by antibiotic class

Antibiotics were prescribed during 1,162 of 10,210 unweighted AGE visits during 2006–2015 (Table 1 and Supplementary Table 3 online). These data equate to a weighted percentage of 13.3% (99% confidence interval [CI], 11.2%–15.4%) and, nationally, an estimated 11.1 million AGE visits (99% CI, 8.9–12.3 million visits) with antibiotics prescribed during the 10-year period.

**Table 1.** Patient Demographics, Clinical Characteristics, and Antibiotic Prescribing Among Acute Gastroenteritis (AGE) Visits in the United States—NAMCS/NHAMCS, 2006–2015

Variable	No. of Unweighted AGE Visits	No. of Unweighted AGE Visits With Antibiotics Prescribed	Weighted AGE Visits With Antibiotics Prescribed, No. (99% CI)^a^	Weighted Mean Percentage of AGE Visits With Antibiotics Prescribed, % (99% CI)^a^	*P* Value^b^
Overall	10,210	1,162	11,074,881 (8,878,806–13,270,956)	13.3 (11.2–15.4)	
**Age**					<.001
0–4 y	2,314	184	2,329,938 (1,342,217–3,317,660)	11.4 (7.1–15.7)	
5–17 y	1,388	97	880,544 (433,665–1,327,422)	7.1 (3.8–10.4)	
18–49 y	3,974	519	4,831,028 (3,427,511–6,234,545)	16.6 (12.6–20.6)	
50–64 y	1,231	168	1,380,939 (848,417–1,913,461)	12.7 (8.1–17.3)	
≥65 y	1,303	194	1,652,432 (1,039,422–2,265,441)	15.7 (10.7–20.7)	
**Sex**					.69
Female	5,985	683	6,337,860 (4,773,197–7,902,523)	13.0 (10.3–15.7)	
Male	4,225	479	4,737,021 (3,319,038–6,155,005)	13.7 (10.2–17.1)	
**Race/Ethnicity**					.57
White, non-Hispanic	6,061	720	7,021,469 (5,415,675–8,627,264)	13.3 (10.8–15.9)	
Black, non-Hispanic	1,914	207	1,407,692 (912,445–1,902,940)	11.3 (7.8–14.9)	
Hispanic	1,733	194	2,198,145 (1,114,349–3,281,941)	14.9 (8.8–21.0)	
Other	502	41	*	*	
**Insurance type**					.53
Medicare/Medicaid	4,051	428	3,545,207 (2,329,263–4,761,151)	12.2 (8.4–15.9)	
Private	3,001	376	4,638,781 (3,372,562–5,905,000)	14.6 (11.2–18.0)	
Other	1,101	125	941,339 (464,520–1,418,158)	13.6 (7.5–19.7)	
Unknown	2,057	233	*	*	
**Practice setting**					<.001
Emergency department	8,770	968	4,452,139 (3,733,274–5,171,004)	10.8 (9.5–12.1)	
Office settings	1,440	194	6,622,742 (4,627,542–8,617,943)	15.7 (11.8–19.7)	
**ICD-9-CM diagnostic code** ^c^					.005
Vomiting without diarrhea	5,767	493	3,579,995 (2,530,484–4,629,505)	10.7 (7.8–13.5)	
Diarrhea without vomiting	2,496	370	4,743,624 (3,356,689–6,130,559)	16.4 (12.3–20.6)	.004
Vomiting with diarrhea	1,430	150	845,295 (568,847–1,121,742)	10.3 (7.3–13.4)	.03
Bacterial gastroenteritis	145	41	*	*	*
**Fever (temperature≥38.3°C or 100.9°F) at the visit**					.04
Yes	443	85	616,921 (248,649–985,192)	20.4 (9.9–30.9)	
No^d^	9,767	1,077	10,457,960 (8,307,306–12,608,615)	13.0 (10.8–15.2)	
**CBC sent**					.78
Yes	5,347	690	4,297,733 (3,160,066–5,435,401)	13.5 (10.5–16.6)	
No	4,863	472	6,777,148 (4,994,573–8,559,722)	13.1 (10.2–16.0)	

Note. AGE, acute gastroenteritis; ICD-9-CM, *International Classification of Diseases, 9* ^*th*^ *revision, Clinical Modification*; CBC, complete blood count.

a Calculated using complex sample methods and sampled visit weights.

b We compared the weighted mean percentage of visits with antibiotics prescribed between strata using the χ^2^ test.

c See Supplementary Table 1 (online) for ICD-9 codes. *P* values for each row are comparing AGE visits with the listed diagnosis to AGE visits without the diagnosis. Sample sizes were too small to generate reliable estimates for other diagnoses.

d Includes 737 visits with unknown temperature.

*Value does not meet standard of reliability or precision.

Among AGE visits with antibiotics prescribed, the most frequently prescribed antibiotic classes were fluoroquinolones (weighted percentage: 28.7%; 99% CI, 21.1%–36.3%), nitroimidazoles (20.2%; 99% CI, 14.0%–26.4%), penicillins (18.9%, 99% CI, 11.6%–26.2%), and cephalosporins (15.1%; 99% CI, 10.1%–20.2%) (Table 2).

**Table 2.** Frequency of Prescriptions by Antibiotic Type Among Acute Gastroenteritis (AGE) Visits with Antibiotics Prescribed—NAMCS/NHAMCS, 2006–2015 and MarketScan, 2014

Antibiotic Type	NAMCS			MarketScan	
	No. of Unweighted AGE Visits With the Antibiotic Type Prescribed^a^	Weighted AGE Visits With Antibiotic Type Prescribed, No. (99% CI)	Weighted Mean Annual Percentage Among AGE Visits with Antibiotics Prescribed, % (99% CI)	No.	% (95% CI)
Fluoroquinolones	380	3,177,454 (2,209,723–4,145,185)	28.7 (21.1–36.3)	85,763	33.3 (33.1–33.5)
Nitroimidazoles	241	2,237,979 (1,473,010–3,002,948)	20.2 (14.0–26.4)	59,871	23.2 (23.1–23.4)
Penicillins	210	2,096,398 (1,182,024–3,010,772)	18.9 (11.6–26.2)	33,120	12.9 (12.7–13.0)
Cephalosporins	202	1,674,014 (1,020,375–2,327,652)	15.1 (10.1–20.2)	18,315	7.1 (7.0–7.2)
Macrolides	150	1,360,429 (729,746–1,991,113)	12.3 (6.9–17.7)	31,118	12.1 (12.0–12.2)
Trimethoprim/sulfamethoxazole	79	839,561 (358,830–1,320,292)	7.6 (3.5–11.7)	14,492	5.6 (5.5–5.7)
Other^b^	117	1,298,689 (403,468–2,193,909)	11.7 (4.4–19.1)	8,031	3.1 (3.1–3.2)

a In total, 1,162 unweighted AGE visits had antibiotics prescribed.

b Other includes aminoglycosides, carbapenems, tetracyclines, aztreonam, lincosamides, vancomycin, nitrofurantoin, polymyxin, colisimethate, fosfomycin, linezolid, daptomycin, telithromycin, tigecycline, and rifaximin.

#### Patient demographics and visit characteristics

Antibiotic prescribing was most frequent among adults aged 18–49 years (weighted percentage: 16.6%; 99% CI, 12.6%–20.6%) and was least frequent among children aged 5–17 years (7.1%; 99% CI, 21.1%–36.3%) (Table 1). Antibiotic prescribing did not differ according to patient sex, race/ethnicity, or insurance type. Antibiotic prescribing was more frequent among AGE visits in office settings than in emergency departments [weighted percentage: 15.7%; (99% CI, 11.8%–19.7%) vs 10.8% (99% CI, 9.5%–12.1%); *P* < .001].

#### Clinical characteristics and diagnosis

Antibiotic prescribing occurred more frequently for febrile patients (temperature ≥38.3°C or 100.9^o^F) compared with those who were afebrile or had no temperature recorded during the visit [weighted percentage: 20.4% (99% CI, 9.9–30.9%) vs 13.0% (99% CI, 10.8–15.2%); *P* = .04]. Antibiotic prescribing did not differ based on whether a CBC was ordered (Table 1). Antibiotic prescribing was most frequent when the visit was coded for diarrhea without vomiting (weighted percentage: 16.4%; 99% CI, 12.3–20.6%). Antibiotic prescribing occurred ∼10% of the time when the visit was coded for vomiting without diarrhea or for vomiting with diarrhea (Table 1).

### Large commercial insurance database estimates (MarketScan, 2014)

#### Prescribing overall and by patient demographics

Of the 1,868,465 MarketScan AGE visits during 2014, antibiotics were prescribed for 13.8% (95% CI, 13.7%–13.8%) (Table 3). Prescribing was most frequent among children 0–4 years old (21.5%; 95% CI, 21.2%–21.7%), and males were most likely to be given an antibiotic prescription (15.7%; 95% CI, 15.6%–15.8%) (Table 3).

**Table 3.** Patient Demographics, Clinical Characteristics, and Antibiotic Prescribing Among Acute Gastroenteritis (AGE) Visits—MarketScan, 2014

Variable	No. of AGE Visits	No. of AGE Visits With Antibiotics Prescribed	Frequency of AGE Visits With Antibiotics Prescribed % (95% CI)
**Overall**	1,868,465	257,731	13.8 (13.7−13.8)
**Age**			
0–4 y	89,242	19,141	21.5 (21.2−21.7)
5–17 y	173,651	24,946	14.4 (14.2−14.5)
18–49 y	1,012,909	134,052	13.2 (13.2−13.3)
50–64 y	592,663	79,592	13.4 (13.3−13.5)
**Sex**			
Female	1,223,508	156,726	12.8 (12.8−12.9)
Male	644,957	101,005	15.7 (15.6−15.8)
**Practice setting**			
Emergency department	600,603	67,428	11.2 (11.2–11.3)
Office settings	811,730	119,896	14.8 (14.7–14.9)
Urgent care center	62,495	11,631	18.6 (18.3−18.9)
Retail clinic	315	51	16.2 (12.1−20.3)
Other/unknown	393,322	58,725	14.9 (14.8–15.0)
**Specialty**			
Emergency medicine	221,920	25,983	11.7 (11.6–11.8)
Family medicine	320,238	53,071	16.6 (16.4–16.7)
Internal medicine	159,651	26,708	16.7 (16.5−16.9)
Gastroenterology	158,864	13,510	8.5 (8.4−8.6)
Pediatrics	87,775	15,037	17.1 (16.9−17.4)
Surgical specialties	13,961	1,538	11.0 (10.5−11.5)
Nurse practitioner/Physician assistant	47,980	7,062	14.7 (14.4−15.0)
Other	1,003,756	128,627	12.8 (12.8–12.9)
**Stool culture order**			
Yes	60,251	18,082	30.0 (29.6−30.4)
No	1,808,214	239,649	13.3 (13.2−13.3)
**ICD-9-CM diagnostic codes^a^**			
Bacterial AGE			
Nontyphoidal *Salmonella*	1,727	548	31.7 (29.5–33.9)
*Campylobacter*	872	391	44.8 (41.5–48.1)
*Escherichia coli*	767	157	20.5 (17.6–23.3)
*Shigella*	619	246	39.7 (35.9–43.6)
Typhoid and paratyphoid fever	275	90	32.7 (27.2–38.3)
*Yersinia*	15	7	46.7 (21.4–71.9)
Unspecified/other bacterial AGE	10,021	2,982	29.8 (28.9–30.7)
Other infectious AGE			
Ill-defined intestinal infections	89,644	27,139	30.3 (29.9−30.6)
Viral gastroenteritis	9,068	1,118	12.3 (11.7−13.0)
AGE symptoms			
Vomiting and diarrhea	183,751	19,926	10.8 (10.7−11.0)
Diarrhea without vomiting	639,603	116,551	18.2 (18.1−18.3)
Vomiting without diarrhea	882,885	87,440	9.9 (9.8−10.0)
Other and unspecified diarrhea from noninfectious causes^b^	50,915	9,870	19.4 (19.0−19.7)

Note. ICD-9-CM, *International Classification of Diseases, 9* ^*th*^ *revision, Clinical Modification.*

a See Supplementary Table 1 (online).

b Not included in AGE definition (see Supplementary Tables 1 and 2 online). Prior studies of hospitalized adults and children with culture-confirmed bacterial enteritis found this code was sometimes used.^13,14^

#### Visit characteristics

Antibiotic prescribing was most frequent in urgent care centers (percentage of visits with antibiotics prescribed: 18.6%; 95% CI, 18.3%–18.9%), followed by office settings (14.8%; 95% CI, 14.7%–14.9%]) and emergency departments (11.2%; 95% CI, 11.2%–11.3%) (Table 3). Antibiotic prescribing was most frequent among providers in primary care specialties (ie, pediatrics, family medicine, internal medicine) (Table 3).

#### Stool culture orders and diagnosis

Among the 257,731 MarketScan AGE visits with antibiotics prescribed, 7.0% had a stool culture order. Antibiotic prescribing was more frequent among visits with a stool culture order at 30.0% (95% CI, 29.6%–30.4%) than those without at 13.3% (95% CI, 13.2%−13.3%) (Table 3).

Visits coded for the following pathogens most frequently had antibiotics prescribed: *Yersinia* (46.7%; 95% CI, 21.4%−71.9%), *Campylobacter* (44.8%; 95% CI, 41.5%−48.1%), *Shigella* (39.7%; 95% CI, 35.9%−43.6%), typhoid or paratyphoid fever (32.7%; 95% CI, 27.2%−38.3%), nontyphoidal *Salmonella* (31.7%; 95% CI, 29.5%–33.9%), *Escherichia coli* (20.5%; 95% CI, 17.6%–23.3%), and unspecified or other bacteria (29.8%; 95% CI, 28.8%–30.7%). Overall, 12.3% (95% CI, 11.7%–13.0%) of visits coded as viral gastroenteritis had antibiotics prescribed. Although not included in our definition of AGE, 19.4% (95% CI, 19.0–19.7%) of visits coded as “other and unspecified diarrhea from noninfectious causes” had antibiotics prescribed (Table 3, Supplementary Tables 1 and 2 online).

Fluoroquinolones were most frequently prescribed for typhoid and paratyphoid fever, nontyphoidal *Salmonella* infections, *E. coli* infections, unspecified or other bacterial AGE, ill-defined intestinal infections, and viral gastroenteritis (Table 4). Macrolides were most frequently prescribed for *Shigella* and *Campylobacter*, and penicillins were most frequently prescribed for *Yersinia* infections.

**Table 4.** Antibiotic Classes Prescribed by Diagnosis Among Acute Gastroenteritis (AGE) Visits with Antibiotic Prescriptions―MarketScan, 2014

Diagnosis and Antibiotic Class	Frequency of Visits With Antibiotics Prescribed, % (95% CI)
**Bacterial AGE**	
**Typhoid and paratyphoid fever (n = 90)**	
Fluoroquinolones	30.0 (20.5−39.5)
Nitroimidazoles	3.3 (0.0−7.0)
Penicillins	13.3 (6.3−20.4)
Cephalosporins	7.8 (2.2−13.3)
Macrolides	24.4 (15.6−33.3)
Trimethoprim/sulfamethoxazole	8.9 (3.0−14.8)
Other	2.2 (0.0−5.3)
**Nontyphoidal** ***Salmonella*** **(n = 548)**	
Fluoroquinolones	42.7 (38.6−46.8)
Nitroimidazoles	6.4 (4.3−8.4)
Penicillins	17.9 (14.7−21.1)
Cephalosporins	7.9 (5.6−10.1)
Macrolides	7.7 (5.4−9.9)
Trimethoprim/sulfamethoxazole	14.1 (11.1−17.0)
Other	1.8 (0.7−3.0)
***Shigella*** **(n = 246)**	
Fluoroquinolones	28.5 (22.8−34.1)
Nitroimidazoles	8.1 (4.7−11.6)
Penicillins	11.4 (7.4−15.4)
Cephalosporins	6.1 (3.1−9.1)
Macrolides	28.9 (23.2−34.5)
Trimethoprim/sulfamethoxazole	15.5 (10.9−20.0)
Other	1.2 (0.0-−2.6)
***Escherichia coli*** **(n = 157)**	
Fluoroquinolones	38.9 (31.2−46.5)
Nitroimidazoles	10.8 (6.0−15.7)
Penicillins	10.8 (6.0−15.7
Cephalosporins	10.8 (6.0−15.7)
Macrolides	8.9 (4.5−13.4)
Trimethoprim/sulfamethoxazole	9.6 (5.0−14.2)
Other	5.7 (2.1−9.4)
***Campylobacter*** **(n = 391)**	
Fluoroquinolones	32.5 (27.8−37.1)
Nitroimidazoles	10.5 (7.5−13.5)
Penicillins	0.8 (0.0−1.6)
Cephalosporins	1.3 (0.2−2.4)
Macrolides	52.7 (47.7−57.6)
Trimethoprim/sulfamethoxazole	0.5 (0.0−1.2)
Other	1.5 (0.3−2.8)
***Yersinia*** **(n = 7)**	
Penicillins	42.9 (6.2−79.5)
Cephalosporins	28.6 (0.0−62.0)
Trimethoprim/sulfamethoxazole	28.6 (0.0−62.0)
**Other infectious AGE**	
**Unspecified/other bacterial AGE (n = 2,982)**	
Fluoroquinolones	53.5 (51.7−55.2)
Nitroimidazoles	21.5 (20.0−23.0)
Penicillins	3.8 (3.1−4.4)
Cephalosporins	2.3 (1.7−2.8)
Macrolides	4.2 (3.5−4.9)
Trimethoprim/sulfamethoxazole	7.5 (6.6−8.5)
Other	5.3 (4.5−6.1)
**Ill-defined intestinal infections (n = 27,139)**	
Fluoroquinolones	52.0 (51.4−52.6)
Nitroimidazoles	23.8 (23.3−24.3)
Penicillins	5.2 (4.9−5.5)
Cephalosporins	3.0 (2.8−3.2)
Macrolides	8.0 (7.7−8.3)
Trimethoprim/sulfamethoxazole	5.2 (4.9−5.4)
Other	1.6 (1.4−1.7)
**Viral gastroenteritis (n = 8,193)**	
Fluoroquinolones	25.0 (24.1−26.0)
Nitroimidazoles	10.2 (9.5−10.8)
Penicillins	23.7 (22.8−24.6)
Cephalosporins	12.6 (11.9−13.3)
Macrolides	18.2 (17.4−19.0)
Trimethoprim/sulfamethoxazole	6.6 (6.1−7.2)
Other	1.0 (0.8−1.2)
**Symptoms**	
***Vomiting with diarrhea*** **(n = 19,926)**	
Fluoroquinolones	40.1 (39.4−40.8)
Nitroimidazoles	24.6 (24.0−25.2)
Penicillins	9.9 (9.5−10.3)
Cephalosporins	5.8 (5.5−6.1)
Macrolides	9.3(8.9−9.7)
Trimethoprim/sulfamethoxazole	6.4 (6.1−6.7)
Other	1.7 (1.5−1.9)
**Diarrhea without vomiting (n = 116,551)**	
Fluoroquinolones	34.5 (34.3−34.8)
Nitroimidazoles	34.5 (34.2−34.8)
Penicillins	7.3 (7.2−7.5)
Cephalosporins	3.8 (3.7−4.0)
Macrolides	8.7 (8.6−8.9)
Trimethoprim/sulfamethoxazole	4.4 (4.3−4.5)
Other	4.5 (4.4−4.6)
**Vomiting without diarrhea (n = 87,440)**	
Fluoroquinolones	25.6 (25.3−25.9)
Nitroimidazoles	9.4 (9.2−9.5)
Penicillins	22.0 (21.8−22.3)
Cephalosporins	12.4 (12.2−12.7)
Macrolides	17.6 (17.3−17.8)
Trimethoprim/sulfamethoxazole	7.1 (6.9−7.3)
Other	2.1 (2.0−2.2)
**Other**	
Other and unspecified diarrhea from noninfectious causes* (n = 9,870)	
Fluoroquinolones	45.9 (44.9−46.9)
Nitroimidazoles	36.1 (35.2−37.1)
Penicillins	4.7 (4.2−5.1)
Cephalosporins	2.5 (2.2−2.8)
Macrolides	3.8 (3.4−4.1)
Trimethoprim/sulfamethoxazole	4.2 (3.8−4.6)
Other	1.9 (1.6−2.2)

Note. Percentages will not sum to 100 because some patients received >1 type of antibiotic.

*Not included in AGE definition (see Supplementary Tables 1 and 2 online). Prior studies of hospitalized adults and children with culture-confirmed bacterial enteritis found this code was sometimes used.^12,13^

#### Antibiotic concordance with 2017 IDSA guidelines

Among AGE visits with antibiotic prescriptions, antibiotics were concordant with 2017 IDSA guidelines for 85.2% (95% CI, 81.6%−88.7%) of visits for *Campylobacter* infection (ie, macrolides or fluoroquinolones) and 84.4% (95% CI, 77.0%−91.9%) of visits for typhoid and paratyphoid fever (ie, cephalosporins, fluoroquinolones, penicillins, macrolides, or trimethoprim-sulfamethoxazole). For *Shigella,* the lower estimate of concordance was 63.4% (95% CI, 57.4%–69.4%) (ie, macrolides, fluoroquinolones, or cephalosporins) and the upper estimate was 90.2 (95% CI, 86.5%–94.0%) (ie, macrolides, fluoroquinolones, cephalosporins, trimethoprim-sulfamethoxazole, or penicillins). Visits for *Yersinia* infection were too uncommon to evaluate concordance.

## Discussion

Using national databases of ambulatory medical visits, we found that ∼13% of AGE visits had an antibiotic prescribed. AGE visits in medical offices and urgent-care centers were significantly more likely to have antibiotics prescribed than those in emergency departments. The most frequently prescribed antibiotic classes were fluoroquinolones, nitroimidazoles, and penicillins. Antibiotics were most often prescribed for visits coded for a bacterial infection or when visit features suggested a bacterial infection. Antibiotics were often prescribed for conditions that do not generally require antibiotics, including *E. coli* infections^15,16^ (21%) and viral gastroenteritis (12%). Antibiotic prescribing occurred at only one-third of visits coded as typhoid or paratyphoid fever, despite antibiotics being indicated for these infections. Antibiotics prescribed were most often concordant with current IDSA guidelines but varied by pathogen. Our findings underscore the need for antibiotic stewardship assessments and interventions for AGE in ambulatory settings.

We identified antibiotic prescribing patterns that were discordant with clinical guidelines. IDSA recommends empiric antibiotic therapy for diarrhea when clinical features of bacterial enteritis are present in certain hosts.^5^ Although vomiting is an uncommon clinical manifestation of bacterial enteritis, antibiotics were prescribed for ∼10% of NAMCS/NHAMCS visits coded for vomiting without diarrhea, or ∼3.6 million visits over the 10-year study period. Similarly, antibiotics were prescribed for 12% of MarketScan visits coded as viral gastroenteritis.

Consistent with previously published data, antibiotic prescribing was higher among visits for illnesses with features more suggestive of bacterial enteritis than those without,^7^ including fever, diarrhea without vomiting, and visits with a stool culture order. Overall, prescribing was less frequent than has been reported previously; a small, prospective study published in 2003 found 93% of patients with presumed bacterial enteritis were prescribed antibiotics.^7^ However, antibiotic prescribing was higher than optimal (13%) for visits without a stool culture order. Although stool cultures are not universally indicated in the evaluation of infectious diarrhea, antibiotic treatment without a stool culture is problematic because it eliminates the ability to choose antibiotics based on culture and antibiotic susceptibility results. Resistance to commonly prescribed antibiotics is increasing among bacterial enteric pathogens.^6,17^

Antibiotic prescribing was relatively high for visits coded for bacterial infections that usually do not require antibiotics (eg, *Yersinia*, 46.7%). Prescribed antibiotics were sometimes discordant with recommended antibiotics based on 2017 IDSA guidelines, although our analysis does not include data after these were published. Clinical trials have not shown that antibiotic therapy shortens the duration of illness for noninvasive *Yersinia* infection.^18^ Penicillins, which are not recommended for *Yersinia* infection, were the most frequently prescribed antibiotic class.^19^ Antibiotic prescribing was also high for nontyphoidal *Salmonella* infection (30.7%) and *E. coli* infection (25.8%). Antibiotics can prolong *Salmonella* shedding^20,21^ and are generally only recommended for patients at increased risk of invasive disease. Antibiotics are generally contraindicated for diarrheal illness caused by *E. coli*, aside from enterotoxigenic *E. coli*, because they can increase life-threatening complications of STEC infection.^15,16^

Prescribing was relatively low for typhoid and paratyphoid fever, which do require antibiotics. Because the MarketScan analysis captures outpatient visits without an associated hospitalization, we may have selected for relatively mild cases of typhoid and paratyphoid fever or gastrointestinal carriage; such cases may have been more prone to delayed antibiotic prescribing (ie, outside the specified 3-day window).

Antibiotic misuse can cause allergic reactions, dysbiosis, and *Clostridioides difficile* infection, and contribute to the development of antibiotic resistance.^22^ Fluoroquinolones were the most frequently prescribed antibiotic class overall and for viral gastroenteritis, prescribed for an estimated 3.2 million visits during the 10-year study period. Fluoroquinolones have been associated with serious adverse events, including tendon rupture and cardiac conduction abnormalities.^23^ Providers prescribing antibiotics for AGE should follow treatment guidelines, consider potential risks and benefits to the patient, and tailor antibiotics based on susceptibility testing results.

Although antibiotic stewardship efforts have been successful in hospital settings and for viral respiratory infections,^24–26^ more work is needed for AGE in outpatient settings. In both analyses, prescribing was lowest in emergency departments, despite illness acuity likely being higher. Providers in medical offices and urgent-care centers may especially benefit from further evaluation of antibiotic prescribing practices and antibiotic stewardship related to AGE. Physicians are more likely to prescribe antibiotics for acute diarrhea when they believe patients expect them.^7^ Public health messaging for patients should emphasize that most diarrheal illnesses do not require antibiotics.

NAMCS/NHAMCS and MarketScan findings were generally similar when overlapping analyses were possible. However, AGE visits among children were approximately twice as likely to have an antibiotic prescription in MarketScan compared with NAMCS/NHAMCS (21% vs 11%), although most pediatric gastroenteritis is viral. These data sets differ regarding period, insurance types, geographic catchment, and inclusion of urgent care settings, factors that have previously been associated with antibiotic prescribing.^27–29^ We did not find differences according to insurance type within NAMCS/NHAMCS. MarketScan only includes patients with commercial health insurance and is not nationally representative.

Our analysis relies on billing codes without the possibility to clinically validate AGE diagnoses. Future analyses might explore how well AGE diagnostic codes correspond with clinical diagnoses, including whether this varies according to provider specialty, practice setting, adoption of ICD-10 codes, or availability of culture-independent diagnostic tests. Previous studies of hospitalized adults and children with culture-confirmed bacterial enteritis found that “other and unspecified diarrhea from noninfectious causes” was sometimes used.^13,14^ We excluded this code from our AGE definition because we assumed that it was more likely used for noninfectious than infectious gastroenteritis. Fully elucidating the somewhat high prescribing for this code was beyond the scope of this analysis.

Our findings have several limitations. These older data may not represent recent trends; interpreting NAMCS/NHAMCS results by year is difficult given the wide, overlapping 99% confidence intervals (Supplementary Table 3 online). For both analyses, we made assumptions to assign the primary indication for antibiotic therapy; antibiotics may have been prescribed for conditions other than AGE. We may have underestimated bacterial enteritis diagnoses given that cultures need time to grow and are not perfectly sensitive; we did not have stool culture results. Our study period largely predates uptake of commercial multiplex stool polymerase chain reaction panels with rapid diagnostic capabilities. Antibiotics may have been prescribed before a diagnostic test result was obtained or an ICD-9-CM code was assigned. We could not determine whether antibiotics were indicated because host factors (eg, international travel, immunocompromising conditions) and illness severity could not be reliably determined. MarketScan relies on a convenience sample of insured patients aged <65 years and is not generalizable; assumptions were made to generate “visits” from insurance claims data, practice settings were based on facility codes, and prescriptions were not captured if they were filled >3 days after the visit or if they did not generate an insurance claim (eg, not filled, patient paid out of pocket).

We estimate that 13% of AGE visits nationwide resulted in antibiotic prescriptions. Despite likely lower illness acuity, patients treated for AGE in medical offices and urgent care centers were significantly more likely to be prescribed antibiotics than those treated in emergency departments. Although most visits did not result in inappropriate use of antibiotics, antibiotics were prescribed for viral gastroenteritis and for some bacterial infections for which antibiotics are not recommended. Our findings suggest that antibiotic stewardship assessments and interventions are needed for AGE in ambulatory settings to reduce unnecessary antibiotic use and improve patient care.

## Supplementary material

For supplementary material accompanying this paper visit http://doi.org/10.1017/ice.2021.522. click here to view supplementary material
