# Supplementary material for: Antibiotic prescribing for acute gastroenteritis during ambulatory care visits—United States, 2006–2015
Source: Infect Control Hosp Epidemiol. 2022 Aug 26;43(12):1880–9. doi: 10.1017/ice.2021.522 (PMC9753066; doi:10.1017/ice.2021.522)
Supplement: Supplementary file 1 [file S0899823X21005225sup001.docx]

**Supplemental Table 1:** **International Classification of Diseases, 9^th^ revision, Clinical Modification** (**ICD-9-CM) codes included as acute gastroenteritis (AGE)**

| Condition | ICD-9-CM codes |
| --- | --- |
| Bacterial gastroenteritis | |
| *Campylobacter* | 008.43 |
| *Escherichia coli* | 008.0-008.09, 041.40-041.43 |
| *Salmonella* infections, non-typhoidal | 003.0–003.9 |
| Salmonella infections, typhoidal | 002.0–002.9 |
| Shigellosis | 004.0–004.9 |
| *Yersinia enterocolitica* | 008.44 |
| “Other/unspecified bacterial gastroenteritis” | |
| Other food poisoning, bacterial (Staphylococcal, *Clostridium perfringens*, other *Clostridia, Vibrio parahaemolyticus*, other bacterial food poisoning, food poisoning unspecified bacterial enteritis), excluding *C. difficile** | 005.0, 005.2-005.9 |
| Intestinal infections due to other bacterial organisms (Arizona group, *Aerobacter aerogenes, Proteus, Staphylococcus, Pseudomonas,* Other anaerobes, Other Gram-negative bacteria) | 008.1–008.42, 008.46–008.49; |
| Bacterial enteritis, unspecified | 008.5 |
| Other infectious AGE | |
| Viral gastroenteritis | |
| Enteritis due to specified virus (rotavirus, adenovirus, Norwalk virus, other small round viruses, calicivirus, astrovirus, enterovirus, other viral enteritis) | 008.6–008.69 |
| Other organism, not elsewhere classified | 008.8 |
| “Ill-defined intestinal infections” | |
| Infectious colitis, enteritis, and gastroenteritis; colitis, enteritis and gastroenteritis of presumed infectious origin, infectious diarrhea, diarrhea of presumed infectious origin | 009.0–009.3 |
| Symptoms | |
| Vomiting (with or without nausea) | 787.01, 787.03 |
| Diarrhea | 787.91 |

* *Clostridium difficile* (008.45) was counted as a tier 1 condition.

**Supplemental Table 2: Tiered diagnoses and corresponding International Classification of Diseases, 9^th^ revision, Clinical Modification** (**ICD-9-CM) codes**^Ɨ^

| Diagnosis | Corresponding ICD-9CM Codes |
| --- | --- |
| **Tier 1 diagnoses: Antibiotics almost always indicated** | |
| *Clostridium difficile* infection* | 008.45 |
| Miscellaneous bacterial infections | 010-018, 020-027, 030-033, 036- 041, 070-104, 130-139, 320-323, 383, 475  Various named infections including tuberculosis, zoonotic bacterial diseases, diphtheria, pertussis, rickettsial and venereal diseases, meningitis, mastoiditis, peritonsillar abscess |
| Pneumonia | 481 Pneumococcal pneumonia [*Streptococcus pneumoniae* pneumonia], 482 Other bacterial pneumonia, 483 Pneumonia due to other specified organism, 484 Pneumonia in infectious diseases classified elsewhere, 485 Bronchopneumonia, organism unspecified, 486 Pneumonia, organism unspecified |
| Urinary tract infections (UTI) | 590.1, 590.2, 590.8, 590.9, 595.0, 595.9, 599.0: Acute pyelonephritis, renal abscess, other pyelonephritis/pyelonephrosis, kidney infection (unspecified), acute cystitis, cystitis (unspecified), UTI (unspecified) |
| **Tier 2 diagnoses: Antibiotics may be indicated** | |
| Acne | 706.0, 706.1  Acne, including vulgaris and varioloformis |
| AGE | See supplemental table 1 |
| Other gastrointestinal infections | 558.9, 001, 007  Other and unspecified noninfectious gastroenteritis and colitis**, cholera, intestinal protozoa |
| Pharyngitis | 462 Acute pharyngitis, 463 Acute tonsillitis, 034 Streptococcal sore throat and scarlet fever |
| Sinusitis | 461 Acute sinusitis, 473 Chronic sinusitis |
| Skin, cutaneous and mucosal infections | 680-686, 035, 110-111, 704.8, 728.0, 611.0, 771.5, 728.86, 380.0-380.1 Includes cellulitis, carbuncle/furuncle, impetigo, lymphadenitis, erysipelas, dermatophytosis, folliculitis, myositis, mastitis, necrotizing fasciitis, infective otitis externa |
| Acute otitis media | 382 Suppurative and unspecified otitis media |
| **Tier 3 diagnoses: Antibiotics not indicated** | |
| Asthma, allergy | 493 Asthma, 477 Allergic rhinitis, 995.3 Allergy, unspecified |
| Bronchitis, bronchiolitis | 490 Bronchitis, not specified as acute or chronic, 466 Acute bronchitis and bronchiolitis, **Excludes visits in which the 2nd or 3rd diagnosis was chronic bronchitis (491), emphysema (492), or COPD (496). |
| Influenza | 487 Influenza, 488 Influenza due to identified avian influenza virus |
| Miscellaneous other infections | 042, 045-049, 050-059, 060-069, 112-129: Human immunodeficiency virus (HIV) infection, poliomyelitis and other non-arthropod-borne viral diseases and prion disease of central nervous system, viral disease accompanied by exanthema, mycoses, helminthiases, arthropod-borne viral diseases |
| Non-suppurative otitis media | 381: Non-suppurative otitis media and Eustachian tube disorders |
| Other gastrointestinal conditions | 520-558.3, 560-579 Other conditions of the digestive system, not already included under gastrointestinal infections |
| Other skin, cutaneous and mucosal conditions | 690-698, 700-709, 870-897, 910-919, 940-949, 360-379, 380-389 (excluding 380.0-380.1, 381, 382, 383), 782, 785.4, 785.6: Skin, cutaneous and mucosal conditions not already categorized as skin, cutaneous and mucosal infections: includes inflammatory and other skin conditions, open wounds, superficial injuries, burns, diseases of the eye/adnexa, ear diseases other than otitis media and mastoiditis, symptoms of skin/integumentary tissues (excluding acne), gangrene, enlargement of lymph nodes |
| Other genitourinary conditions | 580-629, 788.1 (excluding UTI codes above and 611.0): Other genitourinary codes not already categorized as UTI, Other conditions of the genitourinary system, symptoms involving urinary system (including dysuria) |
| Viral pneumonia | 480: Viral pneumonia |
| Viral upper respiratory infection (URI) | 460: Acute nasopharyngitis [common cold], 464 Acute laryngitis and tracheitis, 465 Acute upper respiratory infections of multiple or unspecified sites, 786.2 Cough |
| Other respiratory conditions | All remaining respiratory conditions (460-519) not coded above and 786.0-786.1, 786.3-786.4: Includes chronic bronchitis and bronchitis with a 2nd or 3rd diagnosis of chronic bronchitis, emphysema and COPD; other respiratory conditions; dyspnea; stridor; hemoptysis; abnormal sputum |
| All other codes not listed elsewhere | All remaining codes not listed elsewhere |

^Ɨ^Adapted from eTable 2 in Fleming-Dutra KE, Hersh AL, Shapiro DJ, et al. Prevalence of inappropriate antibiotic prescriptions among US ambulatory care visits, 2010-2011. *JAMA*. 2016.

*Now termed *Clostridioides difficile* infection.

**Supplemental Table 3. Antibiotic prescribing among acute gastroenteritis (AGE) visits in the United States by year—NAMCS/NHAMCS, 2006–2015**

| Year | Unweighted AGE visits, N | Unweighted AGE visits with antibiotics prescribed, N | Weighted mean percentage of AGE visits with antibiotics prescribed, % (99% CI) |
| --- | --- | --- | --- |
| 2006 | 581 | 61 | 16.7 (7.0–26.4) |
| 2007 | 968 | 120 | 9.7 (5.1–14.2) |
| 2008 | 974 | 112 | 16.1 (9.6–22.5) |
| 2009 | 1,069 | 131 | 14.2 (7.4–21.0) |
| 2010 | 1,120 | 126 | 10.6 (5.3–15.9) |
| 2011 | 972 | 99 | 12.7 (5.7–19.7) |
| 2012 | 1,266 | 145 | 11.0 (7.5–14.5) |
| 2013 | 1,163 | 132 | 12.7 (8.4–17.0) |
| 2014 | 1,209 | 138 | 12.1 (7.8–16.4) |
| 2015 | 888 | 98 | 17.2 (7.0–27.4) |
